# Extended In Vitro Maturation Reveals a Prolonged Nuclear Maturation Window and Dynamic BAX/BCL-2 Regulation in Dromedary Camel Oocytes

**DOI:** 10.3390/vetsci13101074

**Published:** 2026-10-11

**Authors:** Waad H. Alhobish, Muath Q. Al-Ghadi, Nouf M. Alyami

**Affiliations:** Zoology Department, College of Science, King Saud University, P.O. Box 2455, Riyadh 11451, Saudi Arabia

**Keywords:** *Camelus dromedarius*, oocyte maturation, BAX/BCL-2 balance, apoptosis, artificial activation, developmental competence

## Abstract

Temporal changes in nuclear maturation, *BAX/BCL-2* transcriptional dynamics, and functional competence during extended in vitro maturation (IVM) remain poorly characterized in dromedary camel oocytes. This study investigated nuclear maturation dynamics, *BAX* and *BCL-2* expressions, and early cleavage competence during extended IVM. Oocytes were evaluated at 0, 24, 30, 36, and 48 h for nuclear maturation, while transcript abundance was analyzed by reverse-transcription quantitative PCR (RT-qPCR) using three independent biological replicates per time point. Early cleavage following artificial activation was compared between 36 and 48 h. The proportion of metaphase II (MII) oocytes increased from 36.36% at 30 h to 69.36% at 36 h and 84.27% at 48 h, with no significant difference between 36 and 48 h. At 24 h, both *BAX* and *BCL-2* were markedly reduced, but the greater reduction in *BCL-2* produced the highest relative *BAX/BCL-2* balance. Between 24 and 30 h, *BCL-2* increased markedly and the relative *BAX/BCL-2* balance declined sharply, remaining low at 36 and 48 h. Cleavage rates were 10.05 ± 0.92% and 12.81 ± 3.45% at 36 and 48 h, respectively, with no significant difference (*p* = 0.299). These findings identify an extended nuclear-maturation window spanning 36–48 h, accompanied by distinct changes in *BAX/BCL-2* transcriptional balance without a detectable reduction in early cleavage competence.

## Simple Summary

Dromedary camel embryo production depends partly on obtaining mature, developmentally competent oocytes under laboratory conditions, but the time required for optimal oocyte maturation in this species remains unclear. This study examined how extending the maturation period affects nuclear maturation, changes in two genes involved in the regulation of cell survival and death, and the ability of oocytes to begin early development after artificial activation. Camel oocytes showed a relatively broad advanced nuclear-maturation window between 36 and 48 h. The balance between the two examined genes changed markedly over time, particularly between 24 and 30 h, but these molecular changes were not shown to be directly associated with nuclear maturation. Oocytes matured for 36 or 48 h also showed similar ability to undergo early cleavage after artificial activation. These findings indicate that dromedary camel oocytes have extended and distinct maturation kinetics and that maturation schedules developed for other domestic species should not be directly applied to camels. The results may help refine laboratory maturation procedures for camel embryo production, although further studies are needed to determine the maturation period that best supports fertilization and later embryo development.

## 1. Introduction

The dromedary camel (*Camelus dromedarius*) is an economically important livestock species in arid and semi-arid regions, particularly in the Middle East. Despite its adaptation to harsh environments, advances in camel reproductive biotechnology remain limited compared with other domestic species. Assisted reproductive technologies (ARTs), including in vitro fertilization (IVF), embryo transfer, gamete cryopreservation, and intracytoplasmic sperm injection (ICSI), have therefore received increasing attention to improve reproductive efficiency and preserve valuable genetic resources [1]. Nevertheless, in vitro embryo production (IVEP) in camels remains relatively inefficient because of biological and technical constraints, including factors affecting oocyte maturation and developmental competence [2,3].

In vitro maturation (IVM) is a fundamental step in embryo production, enabling immature oocytes to acquire the nuclear and cytoplasmic competence required for subsequent development. Nuclear maturation involves meiotic resumption and progression from the germinal vesicle stage to metaphase II (MII), whereas cytoplasmic maturation involves molecular, biochemical, and organelle changes required for fertilization and early embryonic development [4,5]. Coordination of these processes is essential for oocyte competence, as disruption of either may compromise fertilization and embryo development [6,7].

Oocyte quality is a major determinant of embryo production and may deteriorate when maturation or the interval before fertilization is prolonged. In mammalian oocytes, post-maturation aging occurs when mature oocytes remain unfertilized beyond their optimal developmental window and is associated with declining cellular integrity and developmental competence [8]. Prolonged culture and delayed fertilization have also been associated with spindle abnormalities, chromosomal instability, mitochondrial dysfunction, and impaired embryo development [9,10]. However, the temporal boundary between continued maturation and deterioration may vary among species and remains poorly characterized in the dromedary camel.

Oxidative stress and apoptosis are among the cellular processes associated with prolonged oocyte culture and post-maturation aging. Excessive reactive oxygen species (ROS) can cause oxidative damage, disrupt mitochondrial function, and compromise oocyte developmental potential [11]. The BCL-2 protein family plays a central role in mitochondrial apoptotic signaling, with BAX promoting pro-apoptotic signaling and BCL-2 exerting an opposing anti-apoptotic function. Their relative expression patterns may therefore provide insight into the balance of apoptosis-related components during oocyte maturation and prolonged in vitro culture.

Although prolonged oocyte culture and post-maturation aging have been studied in several mammalian species, the molecular changes accompanying extended IVM in dromedary camel oocytes remain poorly understood. Previous camel studies have focused mainly on optimizing maturation conditions and determining the time required for MII attainment, with limited information on apoptosis-related molecular changes and functional competence during extended IVM. Defining their relationship with nuclear maturation is therefore important for understanding the biological limits of camel oocyte IVM and refining in vitro embryo production protocols.

Therefore, the present study aimed to characterize temporal changes during extended in vitro maturation of dromedary camel oocytes by integrating nuclear, molecular, and functional assessments. Specifically, we evaluated nuclear maturation across different IVM durations, temporal changes in *BAX* and *BCL-2* expression and their relative transcriptional balance, and early cleavage competence following artificial activation of oocytes matured for 36 and 48 h. We hypothesized that extended IVM would be accompanied by time-dependent changes in nuclear maturation and *BAX/BCL-2* transcriptional balance, while oocytes within the later maturation window would retain early developmental competence. The findings identify an extended nuclear-maturation window spanning 36–48 h, accompanied by a marked transition in BAX/BCL-2transcriptional balance between 24 and 30 h without a detectable reduction in early cleavage competence.

## 2. Materials and Methods

### 2.1. Chemicals and Reagents

Unless otherwise specified, all reagents and chemicals used in this study were purchased from Sigma-Aldrich (St. Louis, MO, USA).

### 2.2. Experimental Design

A total of 2120 cumulus–oocyte complexes (COCs) recovered from dromedary camel (*Camelus dromedarius*) ovaries were morphologically selected and allocated to five IVM durations: 0, 24, 30, 36, and 48 h. For nuclear maturation assessment, at least 100 oocytes were processed at each time point and distributed among independent experimental replicates. Oocytes with unclear chromosome configurations were excluded from nuclear-stage percentage calculations. Nuclear maturation percentages were calculated separately for each replicate and used for statistical analysis. For gene-expression analysis, 300 oocytes per time point were divided into three independent biological replicates (100 oocytes each), each processed separately for RNA extraction and RT-qPCR. Separate subsets of 60 oocytes per maturation duration were matured for 36 or 48 h and subjected to artificial activation to assess early cleavage competence.

### 2.3. Collection of Ovaries and Oocyte Recovery

Ovaries were collected from freshly slaughtered female dromedary camels at the Central Slaughterhouse in Al-Aziziyah, southern Riyadh, Saudi Arabia (24°34′46.55″ N, 46°44′7.97″ E), and transported to the laboratory within 2–3 h in 0.9% NaCl. Upon arrival, they were washed with warm physiological saline and maintained at approximately 37 °C until oocyte recovery.

COCs were aspirated from 3–8 mm follicles using a 10 mL syringe fitted with a 19-gauge needle containing approximately 0.5 mL of HEPES-buffered TCM-199 (Hank’s solution) supplemented with 10% fetal bovine serum (FBS), 0.3 mM sodium pyruvate, 1 mM L-glutamine, and 25 μg/mL gentamycin sulfate. Follicular aspirates were transferred to 15 mL conical tubes and allowed to sediment for approximately 5 min at 37 °C. The supernatant was removed, and the COC-containing sediment was transferred to Petri dishes containing collection medium for morphological evaluation.

### 2.4. Oocyte Selection

Recovered COCs were examined microscopically and selected based on cumulus investment and cytoplasmic morphology. Only COCs with at least four compact cumulus-cell layers and oocytes with homogeneous cytoplasm were selected, whereas denuded or partially denuded oocytes and those with heterogeneous or abnormal cytoplasm were excluded [12].

### 2.5. In Vitro Maturation

Selected COCs were washed three times in maturation medium and cultured in 100 μL droplets (10–20 COCs/droplet) under embryo-tested mineral oil. The maturation medium consisted of HEPES-buffered TCM-199 (Earle’s solution) supplemented with 10% FBS, 0.3 mM sodium pyruvate, 1 μg/mL estradiol-17β, 0.02 IU/mL follicle-stimulating hormone (FSH), 0.023 IU/mL luteinizing hormone (LH), 25 μg/mL gentamycin sulfate, 1 mM L-glutamine, 100 μM cysteamine, 1× insulin–transferrin–selenium (ITS), and 20 ng/mL epidermal growth factor (EGF). COCs were incubated at 38.5 °C in a humidified atmosphere of 5% CO_2_ for 24, 30, 36, or 48 h, whereas 0 h oocytes were processed without IVM.

### 2.6. Assessment of Nuclear Maturation

Nuclear maturation was evaluated at 0, 24, 30, 36, and 48 h. At each time point, cumulus cells were removed using 300 IU/mL hyaluronidase, and denuded oocytes were fixed for 12 h and stained with 1% aceto-orcein for 20 min. Oocytes were mounted on glass slides and examined under a light microscope at ×400 magnification. Nuclear status was classified as germinal vesicle (GV), germinal vesicle breakdown (GVBD), metaphase I (MI), anaphase I (AI), or metaphase II (MII) based on chromatin configuration. The proportion of MII oocytes was used as the principal indicator of completed nuclear maturation.

### 2.7. Preparation of Oocytes for Gene-Expression Analysis

At each designated time point, cumulus cells were completely removed and denuded oocytes were washed three times in phosphate-buffered saline (PBS). Three independent biological replicates (100 oocytes each) were transferred into RNase-free tubes, snap-frozen in liquid nitrogen, and stored at −80 °C until RNA extraction.

### 2.8. RNA Extraction and RT-qPCR

Total RNA was independently extracted from each biological replicate using an RNA extraction kit (ELK Biotechnology, EP014, Wuhan, China) according to the manufacturer’s instructions and stored at −80 °C until analysis. Relative expression of *BAX* and *BCL-2* was determined by one-step reverse-transcription quantitative PCR (RT-qPCR) using a One Step SYBR Green RT-PCR Mix (ELK Biotechnology, EQ007, China) on a ViiA™ 7 Real-Time PCR System (Applied Biosystems, Foster City, CA, USA), with *GAPDH* as the reference gene. Amplification data were analyzed using QuantStudio™ Real-Time PCR Software (version 1.3; Applied Biosystems, Foster City, CA, USA). Primers for *GAPDH*, *BCL-2*, and *BAX* were synthesized by Macrogen Inc. (Seoul, Republic of Korea) (Table 1).

RT-qPCR reactions were prepared in a final volume of 20 µL, containing 10 µL of 2× One Step RT-PCR Mix, 1.5 µL of RT-PCR Enzyme Mix, 0.4 µL each of 10 µM forward and reverse primers (0.2 µM final concentration), 2.5 µL of RNA template, 0.4 µL of 50× ROX reference dye, and 4.8 µL of RNase-free water. Reverse transcription was performed at 50 °C for 15 min, followed by initial denaturation at 95 °C for 2 min and 40 amplification cycles consisting of 95 °C for 10 s and 60 °C for 30 s.

A common calibrator sample was included in each RT-qPCR run to monitor inter-run variation, and technical replicate Cq measurements were averaged within each biological replicate before subsequent calculations. ΔCt and ΔΔCt were calculated as follows:

ΔCt = Ct(target gene) − Ct(*GAPDH*)

ΔΔCt = ΔCt(sample) − mean ΔCt(0 h group)

Relative transcript abundance was calculated using the 2^−ΔΔCt^ method, with the 0 h group as the calibrator. The relative *BAX/BCL-2* transcriptional balance was calculated independently for each biological replicate as relative *BAX* expression/relative *BCL-2* expression. ΔCt-derived values were used for inferential statistical analysis, whereas relative-expression values were used for presentation.

### 2.9. Artificial Oocyte Activation and Embryo Culture

Separate subsets of oocytes matured for 36 and 48 h were artificially activated. After cumulus removal, oocytes were exposed for 5 min to activation medium supplemented with PVA and 5 µM ionomycin, washed, and cultured for 6 h in KSOM medium containing 10 µg/mL cycloheximide at 38.5 °C under 5% CO_2_. Oocytes were then transferred to supplemented KSOM embryo-culture droplets and incubated for 24 h at 38.5 °C under high humidity in 5% CO_2_, 5% O_2_, and 90% N_2_. Early developmental competence was assessed as the proportion of activated oocytes that cleaved to the 2-cell stage.

### 2.10. Statistical Analysis

Statistical analyses were performed using IBM SPSS Statistics, version 29 (IBM Corp., Armonk, NY, USA). Data are presented as mean ± standard deviation (SD), and normality was assessed using the Shapiro–Wilk test. Nuclear-maturation percentages were calculated independently for each experimental replicate and analyzed using one-way analysis of variance (ANOVA) followed by Tukey’s multiple-comparison test. For RT-qPCR, *BAX* and *BCL-2* expression was statistically analyzed using ΔCt values, whereas relative-expression values were used for presentation. The *BAX/BCL-2* transcriptional balance was calculated independently for each biological replicate and analyzed using one-way ANOVA followed by Tukey’s multiple-comparison test. Cleavage rates from three independent experimental replicates at 36 and 48 h were compared using a two-tailed Welch’s independent-samples *t*-test. Statistical significance was set at *p* < 0.05.

## 3. Results

### 3.1. Nuclear Maturation Dynamics

**Figure 1:**
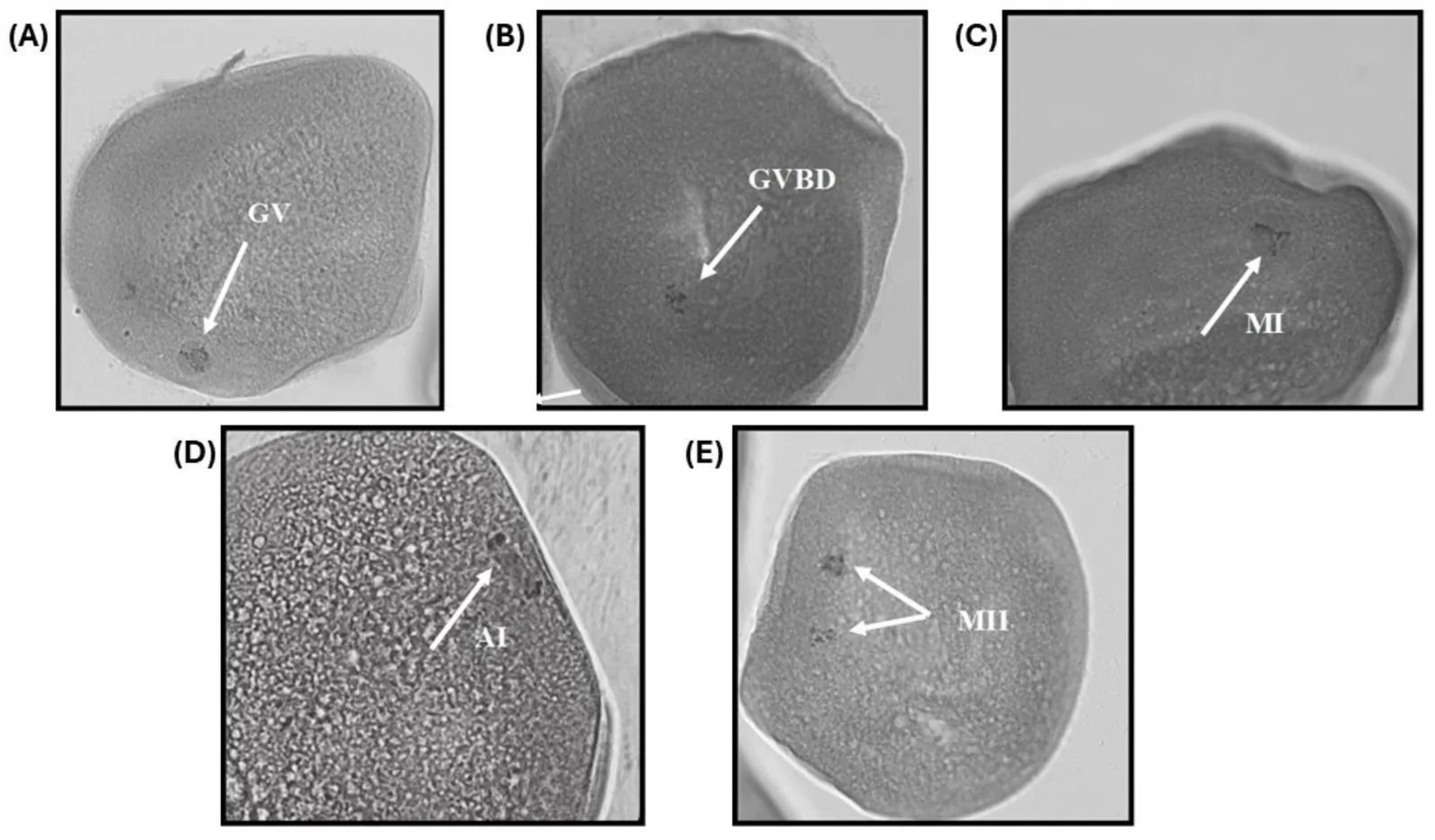
Representative meiotic stages of dromedary camel oocytes during in vitro maturation. Light micrographs showing (**A**) germinal vesicle (GV), (**B**) germinal vesicle breakdown (GVBD), (**C**) metaphase I (MI), (**D**) anaphase I (AI), and (**E**) metaphase II (MII). Arrows indicate the corresponding nuclear or chromosomal configurations.

**Figure 2:**
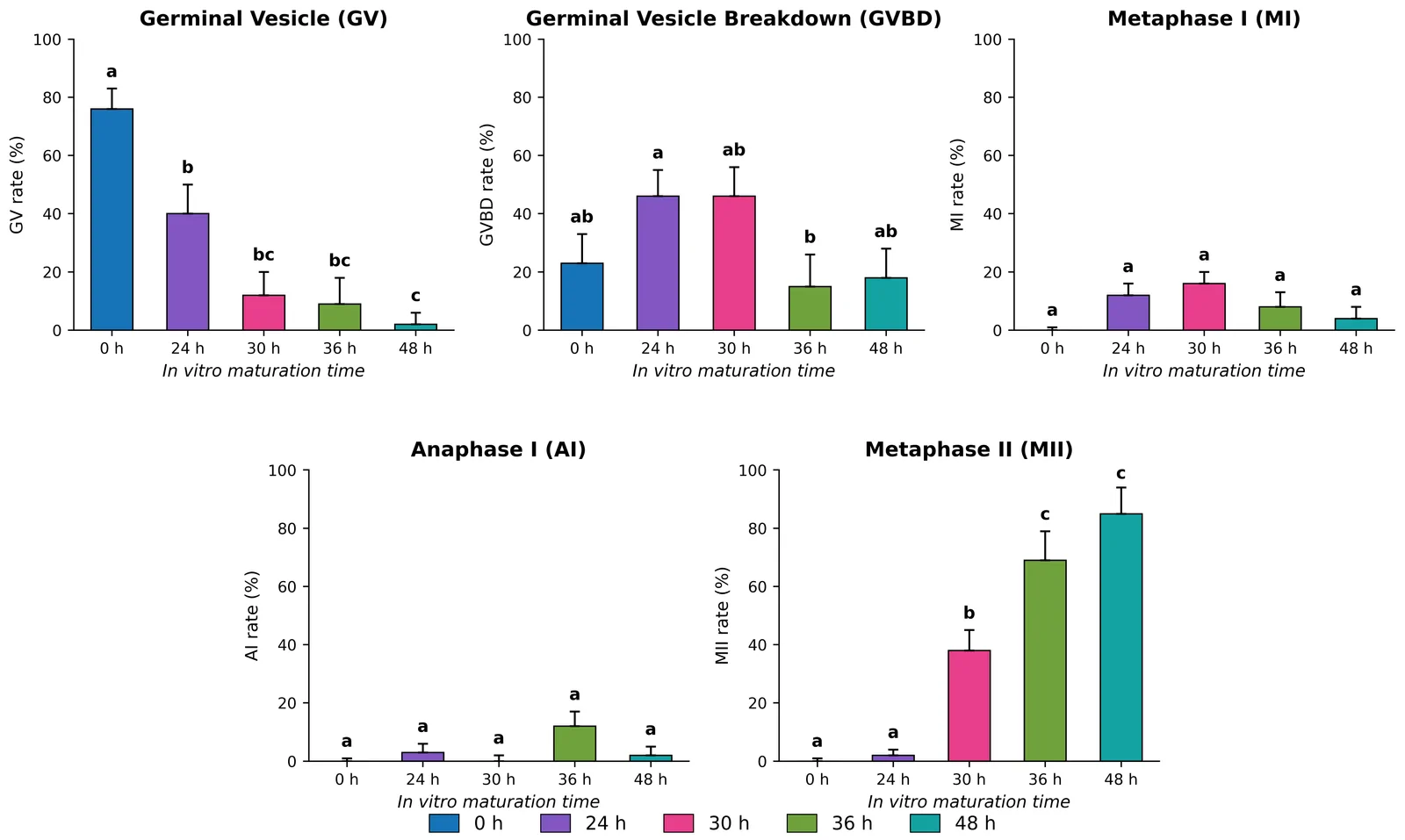
Temporal dynamics of nuclear maturation in dromedary camel oocytes during extended in vitro maturation. Proportions of oocytes at GV, GVBD, MI, AI, and MII after 0, 24, 30, 36, and 48 h of in vitro maturation (IVM). Bars represent mean ± SD. Different lowercase letters within each nuclear stage indicate significant differences among maturation times (one-way ANOVA followed by Tukey’s multiple-comparison test, *p* < 0.05).

IVM duration markedly influenced meiotic progression (Figure 1 and Figure 2). The proportion of oocytes remaining at the GV stage progressively decreased with increasing culture duration, from 77.71% at 0 h to 0.98% at 48 h.

Intermediate meiotic stages were predominantly observed during the early and middle phases of IVM. GVBD was particularly evident during the 24–30 h interval, while MI reached its highest numerical proportion at 30 h (12.12%). These changes were consistent with progressive meiotic resumption rather than accumulation at a single intermediate stage.

The cumulative numbers of classifiable oocytes across the independent experimental replicates were as follows: 86 GV and 22 GVBD at 0 h; 26 GV, 30 GVBD, 8 MI, 3 AI, and 1 MII at 24 h; 6 GV, 25 GVBD, 4 MI, and 24 MII at 30 h; 2 GV, 9 GVBD, 5 MI, 7 AI, and 50 MII at 36 h; and 1 GV, 13 GVBD, 2 MI, 1 AI, and 89 MII at 48 h.

The proportion of MII oocytes increased progressively with IVM duration (Figure 2). MII remained low at 24 h (1.00%), increased to 36.36% at 30 h, and subsequently reached 69.36% at 36 h and 84.27% at 48 h. The difference between 36 and 48 h was not statistically significant (*p* > 0.05).

Thus, although the highest numerical MII proportion was recorded at 48 h, the data identify 36–48 h as an extended nuclear-maturation window rather than demonstrating a statistically superior single optimum at 48 h.

### 3.2. Temporal Dynamics of BAX and BCL-2 Expression

#### 3.2.1. BCL-2 Expression

**Figure 3:**
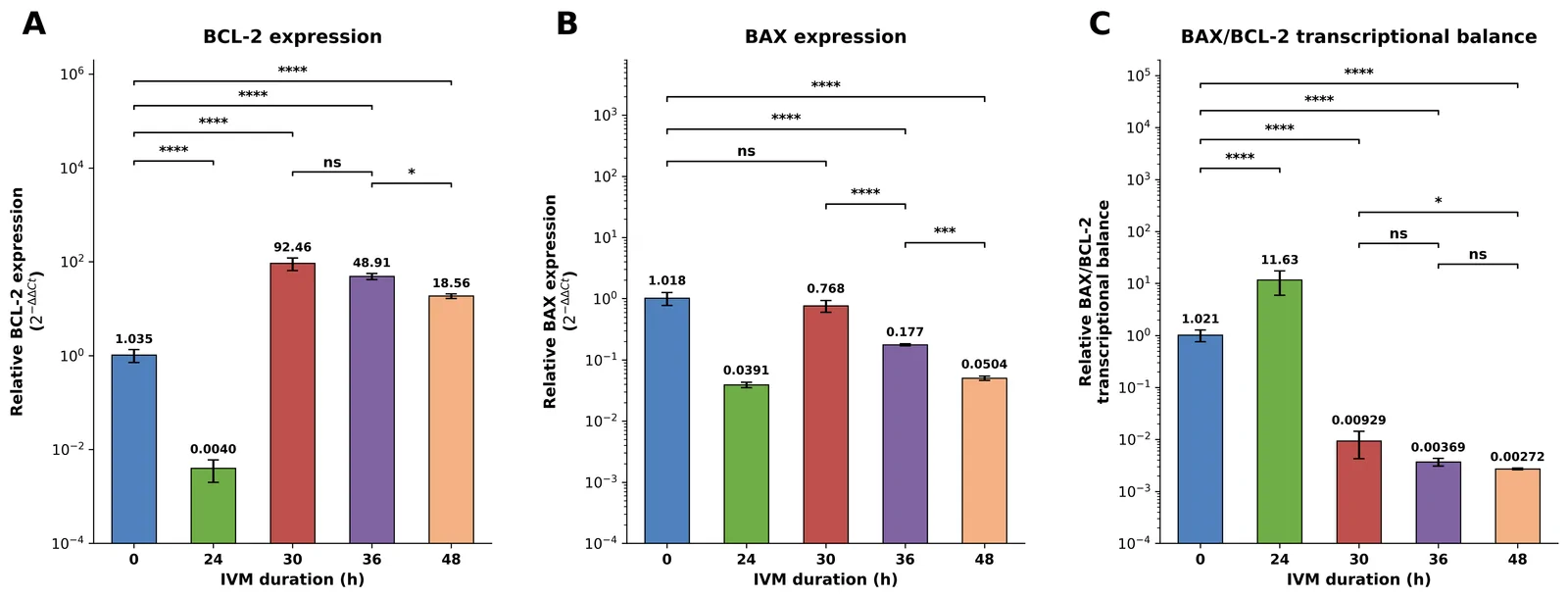
Temporal changes in apoptosis-related transcript abundance during in vitro maturation of dromedary camel oocytes. (**A**) Relative *BCL-2* expression, (**B**) relative *BAX* expression, and (**C**) relative *BAX/BCL-2* transcriptional balance at 0, 24, 30, 36, and 48 h of in vitro maturation (IVM). Data are presented as mean ± SD from three independent biological replicates. Relative *BCL-2* and *BAX* expression was calculated using the 2^−ΔΔCt^ method, with *GAPDH* as the reference gene and the 0 h group as the calibrator. The y-axes are displayed on a logarithmic (base-10) scale. Statistical comparisons were performed by one-way ANOVA followed by Tukey’s multiple-comparison test. * *p* < 0.05, *** *p* < 0.001, **** *p* ≤ 0.0001; ns, not significant.

*BCL-2* transcript abundance differed significantly among IVM durations (one-way ANOVA, *p* < 0.0001; Figure 3A). Expression decreased markedly at 24 h, followed by a sharp increase at 30 h that was maintained through 36 h, with no significant difference between 30 and 36 h (*p* > 0.05). At 48 h, *BCL-2* expression declined significantly compared with both 30 h (*p* < 0.01) and 36 h (*p* < 0.05) (Table 2).

#### 3.2.2. BAX Expression

*BAX* transcript abundance differed significantly among IVM durations (one-way ANOVA, *p* < 0.0001; Figure 3B). Expression decreased markedly at 24 h, returned toward the 0 h level at 30 h without differing significantly from baseline (*p* > 0.05), and subsequently declined significantly between 30 and 36 h (*p* < 0.0001) and between 36 and 48 h (*p* < 0.001) (Table 2).

#### 3.2.3. BAX/BCL-2 Transcriptional Balance

The relative *BAX/BCL-2* transcriptional balance differed significantly among IVM durations (one-way ANOVA, *p* < 0.0001; Figure 3C). The balance increased markedly at 24 h before decreasing sharply at 30 h and remaining very low through 48 h (Table 2). The elevated balance at 24 h reflected a proportionally greater reduction in *BCL-2* than in *BAX*, rather than an absolute increase in *BAX* expression. No significant differences were detected between 30 and 36 h (*p* = 0.0767) or between 36 and 48 h (*p* = 0.8224), whereas 30 and 48 h differed significantly (*p* = 0.0149).

### 3.3. Early Cleavage Competence Following Artificial Activation

**Figure 4:**
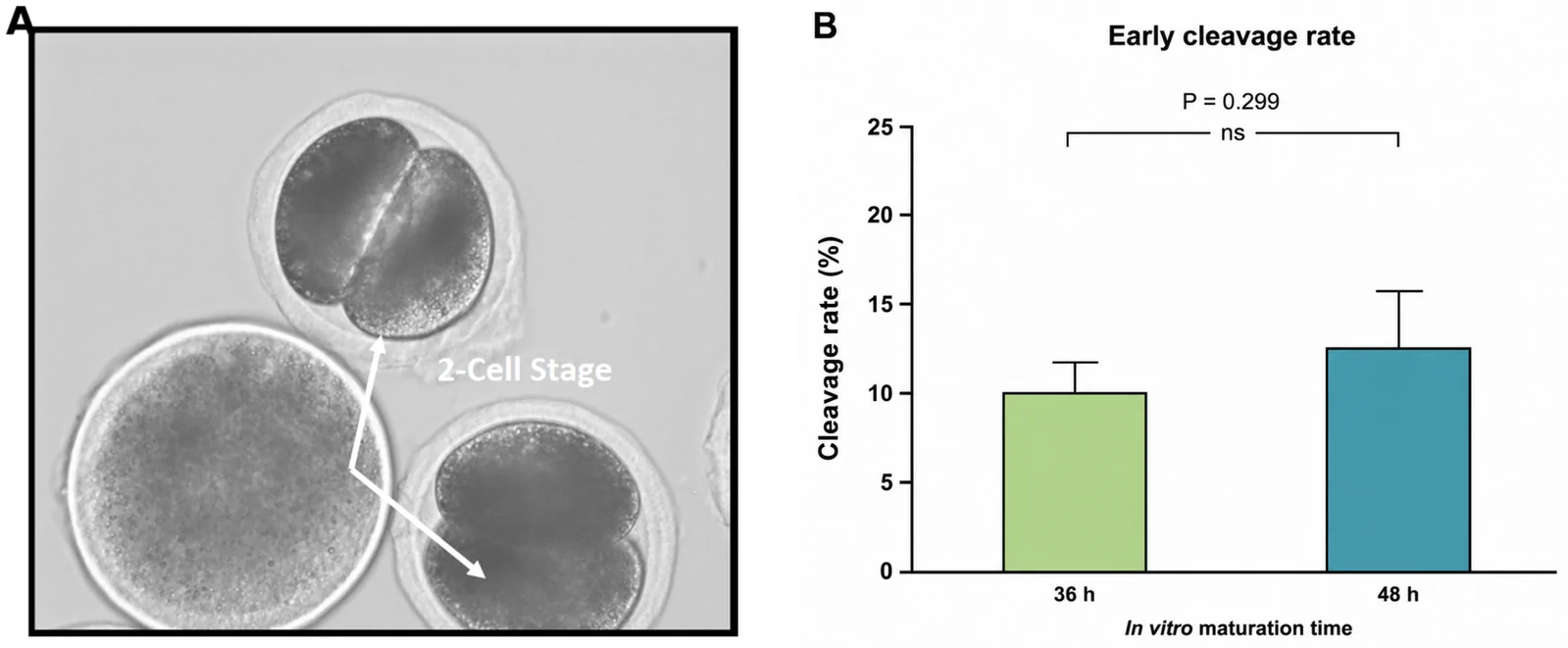
Early cleavage competence of dromedary camel oocytes following artificial activation after extended in vitro maturation. (**A**) Representative image of activated oocytes showing the 2-cell stage. (**B**) Cleavage rates following 36 and 48 h of in vitro maturation (IVM). Data are presented as mean ± SD from three independent experimental replicates. No significant difference was detected between the two maturation durations (two-tailed Welch’s independent-samples *t*-test, *p* = 0.299). In panel (**B**), the green and blue bars represent the 36 h and 48 h IVM groups, respectively.

Oocytes matured for 36 and 48 h were artificially activated to assess early cleavage competence (Figure 4). Cleavage rates were 10.05 ± 0.92% and 12.81 ± 3.45%, respectively. Although numerically higher at 48 h, cleavage did not differ significantly between maturation durations (two-tailed Welch’s independent-samples *t*-test, *p* = 0.299).

## 4. Discussion

The present study provides an integrated characterization of extended IVM in dromedary camel oocytes by examining nuclear maturation, BAX/BCL-2 transcriptional dynamics, and early cleavage competence. Overall, the findings indicate an extended nuclear-maturation window of 36–48 h, accompanied by distinct temporal changes in the BAX/BCL-2 transcriptional balance, while early cleavage competence did not differ significantly between oocytes matured for 36 and 48 h. Together, these findings highlight the extended temporal characteristics of dromedary camel oocyte maturation compared with the shorter maturation schedules commonly reported for other domestic species, while the observed BAX/BCL-2 transcriptional dynamics represent temporally distinct molecular changes rather than evidence of a direct association with meiotic progression.

### 4.1. Nuclear Maturation Dynamics During In Vitro Maturation

Meiotic progression of dromedary camel oocytes was strongly influenced by IVM duration. The decline in GV oocytes, sequential appearance of GVBD and MI stages, and subsequent accumulation at MII reflect progressive meiotic resumption.

MII attainment increased markedly between 30 and 36 h and was numerically highest at 48 h; however, the difference between 36 and 48 h was not significant. This supports an extended nuclear-maturation window of 36–48 h rather than a single statistically superior time point. Wani and Nowshari [13] similarly reported that MII attainment did not differ significantly among 32, 36, 40, 44, and 48 h of IVM, with MII proportions of 42%, 45%, 49%, 52%, and 46%, respectively.

Previous studies have differed regarding the upper limit of the camel oocyte maturation window. Kafi et al. [14] reported MII proportions of 66.5% and 71% after 30 and 42 h of IVM, respectively, compared with 42.5% at 24 h. However, ultrastructural examination revealed signs of cytoplasmic organelle degeneration with prolonged culture, with larger vesicles and lipid droplets becoming evident by 48 h. Abdoon [15] reported that 36 h of IVM produced the highest MII attainment, whereas extending culture to 48 and 72 h significantly increased the proportion of degenerated oocytes. These differences may reflect variation in culture conditions, oocyte source, and follicular characteristics. Therefore, the present findings do not establish 48 h as a universally optimal maturation time, but indicate that extending IVM to 48 h did not compromise nuclear maturation under the conditions of this study.

The extended maturation kinetics of dromedary camel oocytes differ from those of several domestic species. Bovine oocytes, for example, generally reach MII within approximately 20–24 h of IVM [4], whereas camel oocytes require a longer culture period. This species-specific difference indicates that maturation schedules established for other domestic species cannot be directly extrapolated to dromedary camels.

### 4.2. Temporal Dynamics of BAX and BCL-2 Expression During IVM

In addition to nuclear maturation, extended IVM was associated with pronounced temporal changes in *BAX* and *BCL-2* expression. Rather than showing a progressive shift toward a BAX-dominant transcriptional profile, the *BAX/BCL-2* balance changed markedly, particularly between 24 and 30 h. This pattern highlights the importance of considering the relative behavior of both transcripts rather than either gene in isolation.

At 24 h, both *BAX* and *BCL-2* expression decreased markedly relative to baseline, with a substantially greater reduction in *BCL-2*, resulting in an increased BAX/BCL-2 balance. This increase does not reflect absolute *BAX* activation, as *BAX* expression itself remained below baseline, but rather a transient relative imbalance between the two transcripts. Such balance between pro- and anti-apoptotic BCL-2 family members is central to mitochondrial apoptotic regulation [16,17].

The most pronounced molecular transition occurred between 24 and 30 h, when increased *BCL-2* expression and recovery of *BAX* toward baseline were accompanied by a sharp decline in the BAX/BCL-2 balance. This marked transition occurred during the same general period in which meiotic progression was advancing; however, the present data do not establish a direct association between these molecular and nuclear-maturation changes.

BCL-2 expression remained elevated at 36 h and did not differ significantly from 30 h, supporting a high-BCL-2 interval from 30 to 36 h rather than a discrete peak at 36 h. Given the anti-apoptotic role of BCL-2 in mitochondrial apoptosis [16,17], this pattern reflects a low BAX/BCL-2 transcriptional balance during this interval. However, because only transcript abundance was measured, it cannot be interpreted as direct evidence of apoptosis inhibition.

At 48 h, both *BCL-2* and *BAX* expression declined, while the BAX/BCL-2 balance remained very low and did not differ significantly from 36 h. Thus, extended IVM was not accompanied by a progressive shift toward a *BAX*-dominant transcriptional profile. Although oocyte aging is commonly associated with oxidative imbalance, mitochondrial dysfunction, and increased susceptibility to cellular deterioration [8], the transcriptional pattern observed here did not follow a simple progressive pro-apoptotic trajectory.

An important consideration is that transcription becomes largely quiescent in fully grown mammalian oocytes, and meiotic maturation relies substantially on post-transcriptional regulation of maternally stored mRNAs [18,19]. Accordingly, the changes in *BAX* and *BCL-2* transcript abundance observed here are better interpreted as temporal remodeling of the maternal transcript pool rather than direct evidence of transcriptional activation or repression. Whether these changes translate into corresponding alterations in BAX and BCL-2 protein abundance requires direct protein-level analysis.

Overall, these findings demonstrate dynamic temporal changes in the BAX/BCL-2 transcriptional balance during extended IVM, rather than a progressive shift toward a BAX-dominant profile, further emphasizing that the molecular response of dromedary camel oocytes to prolonged maturation is temporally complex.

### 4.3. Integrated Interpretation of Nuclear Maturation, BAX/BCL-2 Transcriptional Balance, and Early Cleavage Competence

When considered together, nuclear maturation and BAX/BCL-2 transcriptional balance exhibited distinct temporal patterns during extended IVM. MII attainment increased during 30–48 h, whereas the BAX/BCL-2 balance remained low over the later maturation period. These parallel temporal observations do not establish a direct association between the two endpoints but indicate that progressive nuclear maturation was not accompanied by increased relative BAX predominance.

Artificial activation provided an additional functional assessment of oocyte competence at the later maturation times. Cleavage competence did not differ significantly between oocytes matured for 36 and 48 h, indicating that extending IVM to 48 h was not associated with a detectable loss of early cleavage competence under the conditions of this study.

Preservation of early cleavage does not necessarily imply equivalent later developmental competence. Yaqoob et al. [20] found that oocytes matured for 32–36 h showed greater embryonic developmental competence following parthenogenetic activation, whereas extending IVM to 42–48 h significantly reduced morula and blastocyst development despite completion of MII. Thus, competence for first cleavage may persist despite declining capacity for later development. Accordingly, the present findings support maintenance of early cleavage competence through 48 h but do not establish equivalent blastocyst-forming capacity.

These findings emphasize that nuclear maturation alone does not fully define oocyte quality. Although MII attainment reflects meiotic competence, it does not necessarily indicate cytoplasmic maturation or subsequent developmental potential [5]. Consideration of nuclear maturation, BAX/BCL-2 transcript profiles, and early cleavage competence therefore provided complementary information on the temporal characteristics of extended dromedary camel oocyte IVM, while each endpoint should be interpreted according to what it directly measures.

### 4.4. Implications for Assisted Reproductive Technologies in Camels

These findings may have practical implications for optimizing camel IVEP protocols. As oocyte maturation remains an important constraint on embryo-production efficiency [3], the extended nuclear-maturation window between 36 and 48 h, together with maintained early cleavage competence at 48 h, suggests some flexibility in the timing of IVM and subsequent laboratory procedures.

Nevertheless, these findings do not establish 48 h as the optimal IVM duration. Because functional competence was assessed only by early cleavage following artificial activation, further studies evaluating fertilization and development to the blastocyst stage are needed to define the optimal IVM interval for camel embryo production. The marked temporal variation in the BAX/BCL-2 balance suggests that BAX/BCL-2 transcriptal patterns may provide complementary molecular information alongside conventional morphological and nuclear assessments of oocyte quality. However, their predictive value remains uncertain and requires validation against fertilization, blastocyst development, and embryo quality.

### 4.5. Limitations and Future Perspectives

The present study has several limitations. Molecular assessment was limited to BAX and BCL-2 transcripts without protein-level analysis or direct measures of apoptosis, mitochondrial function, or oxidative stress. Functional competence was assessed only by early cleavage following artificial activation. Future studies incorporating additional apoptosis-related markers and protein-level analyses, together with direct measures of apoptosis, mitochondrial function, and oxidative stress, as well as fertilization and blastocyst development, are needed to determine the biological significance of the observed BAX/BCL-2 transcriptional changes and their potential relationship with sustained developmental competence.

## 5. Conclusions

The present study demonstrates that dromedary camel oocytes undergo an extended and temporally dynamic maturation process in vitro. Nuclear maturation supported a broad advanced maturation window of 36–48 h, while pronounced changes in the BAX/BCL-2 transcriptional balance occurred primarily between 24 and 30 h. Early cleavage competence did not differ significantly between 36 and 48 h, indicating that extended IVM was not associated with detectable functional deterioration at this early developmental endpoint. These findings characterize distinct temporal patterns in nuclear maturation, BAX/BCL-2 transcriptional balance, and early cleavage competence without establishing a direct association among these endpoints. Further studies incorporating additional apoptosis-related markers, protein-level analyses, and direct measures of apoptosis, fertilization, and blastocyst development are needed to determine the biological significance of these molecular changes and to define the optimal IVM interval for camel embryo production. Overall, the extended maturation kinetics observed in dromedary camel oocytes underscore the species-specific nature of oocyte maturation and indicate that IVM schedules established for other domestic species cannot be directly extrapolated to camels.

## Figures and Tables

**Table 1:** Primer sequences, amplicon sizes, and GenBank accession numbers used for RT-qPCR analysis.

GenBank Accession No.	Amplicon Size (bp)	Sequence (5′-3′) Forward and Reverse	Gene
XM_010990867	134	(F): TGCTGAGTACGTTGTGGAGT (R): TCACGCCCATCACAAACATG	*GAPDH*
XM_010979993.1	92	(F): TGGATCCAGGATAACGGAG (R): TTCAGAGACAGCCAGGAGA	*BCL-2*
XM_010996357.1	190	(F): AGATCATGAAGACAGGGGCC (R): GCGATCATCCTCTGCAACTC	*BAX*

**Table 2:** Relative BCL-2 and BAX expression and BAX/BCL-2 transcriptional balance during extended in vitro maturation.

IVM Time	BCL-2 Relative Expression	BAX Relative Expression	Relative BAX/BCL-2 Transcriptional Balance
0 h	1.035 ± 0.318 ^c^	1.018 ± 0.241 ^a^	1.021 ± 0.259 ^b^
24 h	0.0040 ± 0.0020 ^d^	0.0391 ± 0.0040 ^c^	11.63 ± 5.73 ^a^
30 h	92.46 ± 27.72 ^a^	0.768 ± 0.164 ^a^	0.0093 ± 0.0050 ^c^
36 h	48.91 ± 7.21 ^a^	0.177 ± 0.007 ^b^	0.00369 ± 0.00061 ^cd^
48 h	18.56 ± 2.08 ^b^	0.0504 ± 0.0040 ^c^	0.00272 ± 0.00010 ^d^

Values are presented as mean ± SD (*n* = 3 biological replicates per time point). Within each column, means sharing at least one superscript letter are not significantly different, whereas means with no superscript letters in common differ significantly (*p* < 0.05; one-way ANOVA followed by Tukey’s multiple-comparison test). Relative BCL-2 and BAX expression was calculated using the 2^−ΔΔCt^ method, with the 0 h group as the calibrator. Statistical comparisons were performed using ΔCt-derived values.

## Data Availability

The original contributions presented in this study are included in the article. Further inquiries can be directed to the corresponding author.
